# Evaluation of Fibrinolytic Inhibitors: Alpha-2-Antiplasmin and Plasminogen Activator Inhibitor 1 in Patients with Obstructive Sleep Apnoea

**DOI:** 10.1371/journal.pone.0166725

**Published:** 2016-11-18

**Authors:** Maciej Zakrzewski, Ewelina Zakrzewska, Paweł Kiciński, Sylwia Przybylska-Kuć, Andrzej Dybała, Wojciech Myśliński, Jolanta Pastryk, Tomasz Tomaszewski, Jerzy Mosiewicz

**Affiliations:** 1 Department of Internal Diseases, Medical University of Lublin, Lublin, Poland; 2 Department of Experimental Haematooncology, Medical University of Lublin, Lublin, Poland; 3 Department of Family Medicine, Medical University of Lublin, Lublin, Poland; 4 Clinic of Maxillofacial Surgery, Medical University of Lublin, Lublin, Poland; 5 Department of Pathophysiology, Medical University of Lublin, Lublin, Poland; Nagoya University, JAPAN

## Abstract

Obstructive sleep apnoea (OSA) induces thrombophilia and reduces fibrinolysis. Alpha-2-antiplasmin (a-2-AP) and plasminogen activator inhibitor 1 (PAI-1) are major inhibitors of the fibrinolytic system. Increased concentrations of these factors are associated with a higher risk of cardiovascular diseases. The aim of this study was to assess plasma a-2-AP and PAI-1 in patients with OSA and evaluate correlations with the polysomnographic record and selected risk factors of cardiovascular diseases. The study group comprised 45 patients with OSA, and the control group consisted of 19 patients who did not meet the diagnostic criteria of OSA. Plasma a-2-AP and PAI-1 concentrations were assessed by enzyme-linked immunosorbent assay (ELISA). In the study group, the median value of plasma a-2-AP was higher than that of the control group (157.34 vs. 11.89 pg/ml, respectively, *P*<0.0001). A-2-AP concentration increased proportionally to the severity of OSA. The concentration of a-2-AP was positively correlated with the apnoea-hypopnoea index (AHI), apnoea index (AI), respiratory disturbances time (RDT), and desaturaion index (DI), and negatively correlated with mean and minimal oxygen saturation (SpO_2_ mean, SpO_2_ min, respectively). The median value of PAI-1 was higher in the study group than the control group (12.55 vs. 5.40 ng/ml, respectively, *P* = 0.006) and increased along with OSA severity. PAI-1 concentration was positively correlated with AHI, AI, RDT, DI, and body mass index (BMI) and negatively correlated with SpO_2_ mean and SpO_2_ min. Higher plasma concentrations of a-2-AP and PAI-1 in patients with OSA indicated that these patients had increased prothrombotic activity. OSA increases the risk of cardiovascular complications as it enhances prothrombotic activity.

## Introduction

Obstructive sleep apnoea (OSA) is the most common type of sleep-related respiratory disorder. It is characterized by repetitive pauses in breathing during sleep or considerably reduced air flow despite the effort to breathe normally. Those episodes are defined as apnoeas and hypopnoeas, respectively. OSA is three times more frequent among males than females [1]. The major risk factors of OSA include obesity, age, smoking, type 2 diabetes, and hypothyroidism [2–5].

Numerous clinical studies have found a strong correlation between OSA and cardiovascular diseases. The effects of OSA on hypertension and ventricular or supraventricular arrhythmias have been extensively studied [6–8]. OSA is also an independent risk factor of ischaemic heart disease, stroke or sudden cardiac death [9–14]. Recent studies have focused on the effects of sleep disorders on blood clotting and fibrinolysis. The major function of the fibrinolytic system is to decompose fibrin and maintain blood liquidity. Its components are involved in various processes, including embryogenesis, angiogenesis and wound healing [15]. It may also play a role in pathological processes. Moreover, specific components of the fibrinolytic system have been postulated to be involved in developing atherosclerosis, thrombosis, liver diseases, and oncogenesis [16, 17]. Obstructive sleep apnoea induces thrombophilia and impairs fibrinolysis, which explains the increased risk of cardiovascular disease in these patients [18, 19]. Increased concentrations of plasma clotting factors, such as fibrinogen, factor VII, or factor XII, might be responsible for the enhanced blood clotting [20]. Elevated TAT (thrombin-antithrombin III), a well-known marker of coagulant activity, has also been reported [21].

Alpha-2-antiplasmin (a-2-AP) and plasminogen activator inhibitor 1 (PAI-1) have a crucial role in reducing plasmin production and activity and thus inhibiting fibrinolysis.

Alpha-2-antiplasmin is the major plasma inhibitor of plasmin. It may also inactivate trypsin, elastase, and C protein. It belongs to the serpin family and is synthesized in the liver as a single chain glycoprotein with a molecular weight of 51 kDa. The enzyme is present in two forms. The longer chain contains 464 amino acids and represents approximately 30% of the circulating antiplasmin. The remaining 70% consists of the other form, which has 452 amino acids and higher proteolytic activity [22,23]. Alpha-2-antiplasmin circulates in the blood, both in the free form and bound to plasminogen, and its half-life is 2–6 days [24]. Recent reports have indicated that a-2-AP may be involved in various pathological processes. In addition to its prothrombotic activity, its involvement in carcinogenesis and angiogenesis is being investigated. Many authors have also suggested that the complex PAP (plasmin-a-2-AP) may be an important laboratory marker of haemostasis. As in the case of D-dimer, its increased concentration may indicate enhanced plasminogenesis and thus indirectly reflect the ongoing thrombotic process [25].

Under physiological conditions, PAI-1 is synthesized by hepatocytes, spleen cells, endothelial cells, smooth muscle cells, adipocytes, and blood platelets. Inhibitor activity is increased in cancer, bacterial infections, and other inflammatory processes, e.g., acute pancreatitis [26, 27]. PAI-1 concentration also increases with age. Thus, fibrinolytic activity is reduced in the elderly, which may explain the increased incidence of thrombosis in this age group [28]. PAI-1 binds active molecules of urokinase plasminogen activator (u-PA) and tissue plasminogen activator (t-PA) and forms stable inactive complexes. Adipose tissue appears to be an important source of circulating PAI-1. Its concentration is positively correlated with body mass index (BMI) as well as waist to hip ratio (WHR) [29–31].

The purpose of this study was to analyse the effects of obstructive sleep apnoea on alpha-2-antiplasmin and plasminogen activator inhibitor 1 concentrations and to evaluate the correlations between these parameters with polysomnographic indices and selected risk factors of cardiovascular diseases.

## Material and Methods

### Ethics

This study was approved by the Ethics Committee of Medical University of Lublin, Poland (approval number: KE-0254/124/2012). All patients provided written consent forms approved by the Ethics Committee of Medical University of Lublin, Poland (approval number: KE-0254/124/2012)

### Study group

The study was conducted in 2012–2014 and included 64 adult patients admitted to the hospital for diagnosis of breathing problems during sleep. Patients with a history of cardiovascular diseases (except hypertension), hyperlipidaemia or any condition that may have an impact on coagulation (i.e., malignancies, chronic inflammatory diseases, liver dysfunction, recent surgeries, thrombophilias) were excluded from this study. A history of hypertension, diabetes and obesity was not exclusionary. The study group comprised 45 patients, 41 men and 4 women aged 34–81 years, who were diagnosed with obstructive sleep apnoea. The diagnosis of OSA was confirmed by polysomnography results. This group was divided into 3 subgroups based on the severity of OSA.

The control group consisted of 19 patients, 14 males and 5 females, aged 39–59 years, who did not meet the diagnostic criteria of OSA.

Patients who met the polysomnographic criteria of central sleep apnoea were not eligible for this study.

### Medical history

In the first stage of qualification for the study, each patient's medical history was collected for symptoms suggestive of sleep-related breathing disorders, comorbidities, medication taken, and smoking status. In addition, the patients were surveyed using the Epworth Sleepiness Scale (ESS) to assess the severity of daytime sleepiness and the Berlin Questionnaire (BQ), which is a diagnostic tool to rank the presence and severity of symptoms suggestive of abnormal breathing during sleep.

### Polysomnography

All patients underwent polysomnography using a SleepDoc Porti 6 and Porti 8 (Dr. Fenyves und Gut Deutschland GmbH). To identify breathing disorders during sleep, a polysomnogram was taken at night, from 22:00–23:00 to 05:00 a.m. An event was diagnosed as sleep apnoea when a state of complete or almost complete reduction in the amplitude of breath below 90% and cessation of airflow in the airways for at least 10 seconds occurred. Hypopnoea was considered an episode of reduced airflow by at least 30%, accompanied by desaturation (≥4%) for at least 10 seconds. Desaturation was diagnosed when blood oxygen saturation fell by ≥4% compared to the baseline. To diagnose and grade the severity of obstructive sleep apnoea, the number of apnoeas and hypopnoeas per hour was reported and the apnoea-hypopnoea index (AHI) calculated. OSA was diagnosed when AHI≥5, and clinical symptoms were observed. AHI level was used to divide the study group into 3 subgroups:

1. Mild OSA (5≤AHI<15), 11 patients
2. Moderate OSA (15≤AHI<30), 10 patients
3. Severe OSA (AHI≥30), 24 patients

The following polysomnographic parameters were analysed: apnoea-hypopnoea index (AHI), apnoea index (AI), i.e., the number of apnoeas per hour, desaturation index (DI), i.e., the number of desaturation episodes per hour, minimum oxygen saturation (SpO_2_ min), mean oxygen saturation (SpO_2_ mean), respiratory disturbances time (RDT), i.e., total time of respiratory disorders during examination, and t90, i.e., total time in which saturation dropped below 90%.

### Laboratory tests

All patients had blood samples collected between 6:00 a.m. and 7:00 a.m. on the day after admission to the hospital. Laboratory tests included the following measurements: complete blood count (CBC), creatinine, urea, alanine aminotransferase (ALT) and aspartate aminotransferase (AST), thyroid stimulating hormone (TSH), potassium, sodium, and glucose.

### Plasma alpha-2-antiplasmin and PAI-1 concentration assays

To determine alpha-2-antiplasmin concentration, the blood was collected in tubes containing ethylenediaminetetraacetic acid (EDTA) and in tubes with sodium citrate for the PAI-1 assays. The samples were centrifuged at 4°C for 20 min at 4000 r/min. The obtained serum was stored at -80°C until testing.

The concentrations of alpha-2-antiplasmin and PAI-1 in peripheral blood plasma were determined by enzyme-linked immunosorbent assay (ELISA) using reagent kits:

1. Human Alpha-2-antiplasmin ELISA kit, test sensitivity of 62.5 pg/ml (EIAab Wuhan, China).
2. Human PAI-1 Platinum ELISA, test sensitivity of 29 pg/ml (eBioscence, Vienna, Austria).

The results were automatically analysed by an ELX800 ELISA reader (Biotek Instruments, Winooski, USA). The software for the ELISA reader generated the curves, standard, logarithmic and semi-logarithmic, from the light absorbance of the test wells and wells of known concentrations, and the concentrations of the test samples were calculated.

### Statistical analysis

The results were statistically analysed by Statistica 10.0 PL (StatSoft Inc., USA) and are presented as the median with standard deviation, the minimum value of the statistical series (min), and the maximum value of the statistical series (max). The distribution within groups was assessed using the Shapiro-Wilk test. Because the test variables were not normally distributed, further analysis by non-parametric tests was performed. The Mann-Whitney U test was used to compare the differences between the study and the control groups. The correlations between the variables were assessed using Spearman's rank correlation coefficient. Multiple linear regression analysis was performed to determine the potential independent influence of various factors on a-2-AP and PAI-1 concentration. To compare categorical data, the χ^2^ test was used. The results considered significant at p≤0.05.

## Results

### Clinical symptoms in the study group vs. control

There were no statistically significant differences between the groups in patient’s age (p = 0.15) or systolic (p = 0.72) and diastolic (p = 0.33) blood pressure. BMI was significantly higher in the patients with obstructive sleep apnoea (33.50 vs. 30.90, p = 0.03).

Obesity, hypertension, type 2 diabetes, and smoking were not significantly different between the groups.

Significant differences were found between the groups in polysomnography reports. Table 1 presents the results.

**Table 1:** Polysomnographic parameters in the study and control group.

	Study group AHI≥5 (n = 45)	Control group AHI<5 (n = 19)	
Parameters	Median ± SD		P-value*
AHI (h^-1^)	31.0±21.3 (6.2–88.2)	3.6±1.7 (0–4.8)	<0.0001
AI (h^-1^)	17.8±19.1 (2.2–86.2)	0.8±1.0 (0–3.2)	<0.0001
SpO_2_ mean (%)	91.0±2.3 (83.0–94.0)	92.0±2.1 (87.0–95.0)	0.04
SpO_2_ min (%)	76.0±13.0 (23.0–89.0)	84.0±4.9 (77.0–93.0)	0.00005
t90 (min.)	16.0±19.8 (0–77.0)	2.0±4.8 (0–15.0)	0.0003
RDT (min.)	60.0±70.4 (7.0–266.0)	7.0±5.1 (0–15.0)	<0.0001
DI (h^-1^)	34.7±27.7 (6.5–116.0)	5.6±7.6 (1.0–30.2)	<0.0001

AHI, apnoea-hypopnoea index; AI, apnoea index; DI, desaturation index; RDT, respiratory disturbances time; SpO_2_ mean, mean oxygen saturation; SpO_2_ min, minimum oxygen saturation; t90, time in which oxygen saturation dropped below 90%; SD, standard deviation

* p-value = Mann-Whitney test.

### Plasma alpha-2-antiplasmin and PAI-1 concentrations in the patients with OSA

Plasma a-2-AP in the patients with OSA was significantly higher compared to the control group (157.34 vs. 11.89 pg/ml, p<0.0001). The concentration of a-2-AP increased significantly with increasing degree of OSA severity.

**Fig 1:**
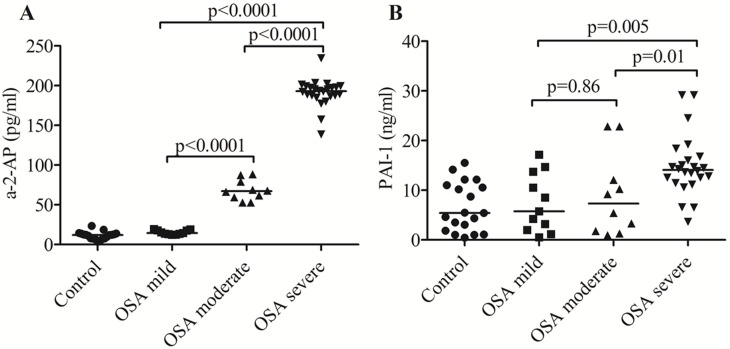
The concentrations of plasma a-2-AP and PAI-1 in the patients with OSA and the control group. The concentrations of alpha-2-antiplasmin and PAI-1 in peripheral blood plasma were determined by enzyme-linked immunosorbent assay (ELISA) in 45 OSA patients and 19 controls. (A) and (B) The concentrations of plasma a-2-AP and PAI-1 in the patients with different grades of OSA severity. Bars represent median value. p values were calculated by the Mann-Whitney test.

In the patients with OSA, plasma PAI-1 concentration was significantly higher compared to the control group (12.55 vs. 5.40 ng/ml, p = 0.006). Plasma PAI-1 concentration in the patients with different degrees of OSA severity was significantly higher in the patients with severe OSA compared with mild OSA (p = 0.005) and with moderate OSA (p = 0.01). No significant differences in plasma PAI-1 were observed between the patients with mild OSA and moderate OSA (*P* = 0.86). (Fig 1), and Table 2 presents the results.

**Table 2:** The concentration of alpha-2-antiplasmin and plasminogen activator 1 in plasma of patients with obstructive sleep apnoea and the control group.

	Control group	OSA	Mild OSA	Moderate OSA	Severe OSA
	Median±SD				
PAI-1 (ng/ml)	1. 5.40±4.92 2. (0.44–15.50)	1. 12.55±7.28 2. (0.51–29.16)	1. 5.75±5.87 2. (0.51–17.14)	1. 7.31±8.27 2. (0.95–22.84)	1. 14.08±6.13 2. (3.66–29.16)
a-2-AP (pg/ml)	1. 11.89±4.82 2. (3.45–23.45)	1. 157.34±79.19 2. (12.11–234.55)	1. 14.54±2.70 2. (12.11–19.45)	1. 67.16±12.85 2. (52.76–88.43)	1. 193.06±17.28 2. (138.59–234.55)

a-2-AP, alpha-2-antiplasmin; OSA, obstructive sleep apnoea; PAI-1, plasminogen activator inhibitor 1; SD, standard deviation.

### Plasma alpha-2-antiplasmin and PAI-1 concentrations vs. age and body mass index in the patients with OSA

There was no significant correlation between plasma a-2-AP and age (r = 0.02; p = 0.86) in the patients with OSA. However plasma a-2-AP was positively correlated with BMI (r = 0.35; p = 0.01).

We found a positive correlation between plasma PAI-1 and BMI in the patients with OSA (r = 0.29; p = 0.04). No significant correlation was found between PAI-1 concentration and age (r = 0.02; p = 0.87).

### Plasma alpha-2-antiplasmin and PAI-1 concentrations vs. hypertension, type 2 diabetes and smoking in patients with OSA

No statistically significant associations were observed between plasma a-2-AP concentrations and hypertension (p = 0.42), type 2 diabetes (p = 0.22), and smoking (p = 0.74).

In the patients with OSA, statistically significant associations were found between plasma PAI-1 concentrations and hypertension (p = 0.02). However, no significant correlations were found between PAI-1 concentrations and type 2 diabetes (p = 0.50) and smoking (p = 0.74).

Table 3 presents the results.

**Table 3:** The alpha-2-antiplasmin and plasminogen activator inhibitor 1 concentrations in the plasma of patients with OSA in relation to selected risk factors of cardiovascular diseases.

	PAI-1 (ng/ml)	a-2-AP (pg/ml)
Hypertension vs Normal blood pressure	12.76±6.13 (3.17–24.50) vs 3.66±3.93 (0.95–10.58)	182.51±73.53 (12.34–203.43) vs 88.43±77.25 (17.89–192.23)
Type 2 diabetes vs Non diabetes	3.31±9.93 (0.95–19.22) vs 10.58±6.30 (1.30–24.50)	67.88±43.43 (59.54–138.59) vs 187.56±77.87 (12.34–203.43)
Smoking vs Non smoking	10.97±7.76 (1.30–24.50) vs 8.16±5.71 (0.95–15.05)	182.55±77.80 (12.34–03.43) vs 186.28±83.08 (17.89–199.22)

a-2-AP, alpha-2-antiplasmin; PAI-1, plasminogen activator inhibitor 1.

### Plasma alpha-2-antiplasmin and PAI-1 concentrations and the polysomnographic parameters AHI, AI, RDT, DI, SpO_2_ mean, SpO_2_ min, and t90 in patients with OSA

In the OSA group, plasma a-2-AP was positively correlated with AHI (r = 0.83; p = 0.0001), AI (r = 0.73; p = 0.0001), RDT (r = 0.76; p = 0.0001), DI (r = 0.77; p = 0.0001), and t90 (r = 0.50; p = 0.0001). However, plasma a-2-AP concentrations were negatively correlated with SpO_2_ mean (r = -0.5; p = 0.0001) and SpO_2_ min (r = -0.58; p = 0.0001).

In the group of patients with OSA, there was a significant positive correlation between plasma PAI-1 concentrations and AHI (r = 0.40; p = 0.006), AI (r = 0.42; p = 0.004), RDT (r = 0.38; p = 0.01), and DI (r = 0.39; p = 0.009). PAI-1 concentrations were negatively correlated with SpO_2_ min (r = -0.34; p = 0.02) and SpO_2_ mean (r = -0.30; p = 0.04). No significant correlation was found between PAI-1 and t90 (r = 0.24; p = 0.1).

**Fig 2:**
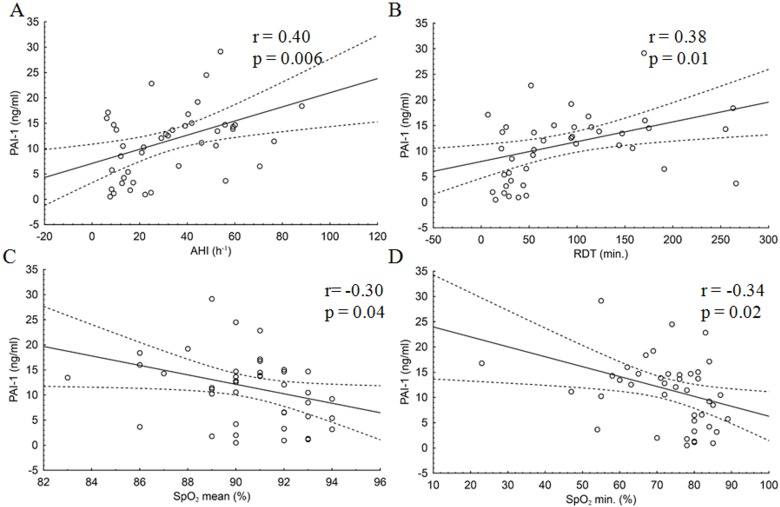
Correlations between selected polysomnographic parameters and PAI-1 concentration in patients with OSA. Breathing disorders during sleep were identified by polysomnography, which was taken at night, from 22:00–23:00 to 05:00 a.m. To diagnose and grade the severity of obstructive sleep apnoea, the number of apnoeas and hypopnoeas per hour was reported and the apnoea-hypopnoea index (AHI) calculated. Correlations between PAI-1 concentrations and polysomnographic parameters: (A) AHI, (B) RDT, (C) SpO_2_ mean, (D) SpO_2_ min. r and p values were calculated by Spearman’s rank order correlation test.

**Fig 3:**
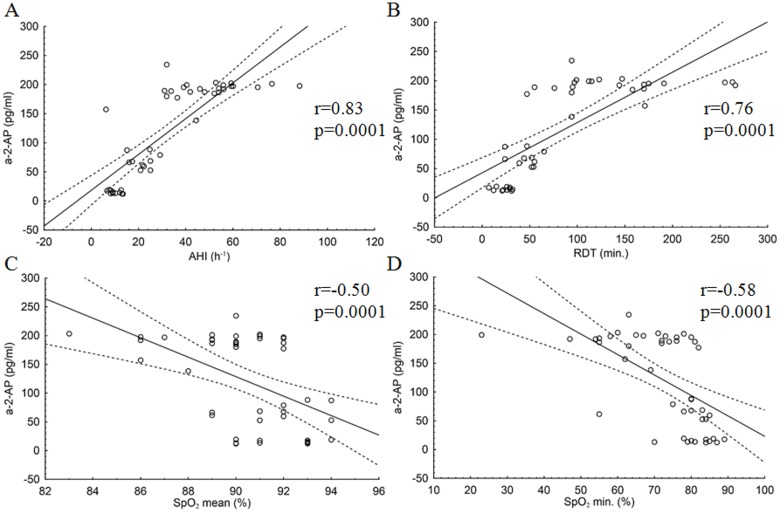
Correlations between selected polysomnographic parameters and a-2-AP concentration in patients with OSA. Correlations between a-2-AP concentrations and polysomnographic parameters: (A) AHI, (B) RDT, (C) SpO_2_ mean, (D) SpO_2_ min. r and p values were calculated by Spearman’s rank order correlation test.

Figs 2 and 3 present the association between selected polysomnographic parameters and a-2-AP and PAI-1 concentrations in patients with OSA.

### Multiple linear regression analysis of the influence of BMI and polysomnographic parameters on alpha-2-antiplasmin and PAI-1 concentrations

The study group had higher AHI, AI, DI, RDT, t90 values as well as BMI and lower levels of SpO_2_ mean and SpO_2_ min than the control group. Significant correlations between most polysomnographic parameters and BMI and a-2-AP and PAI-1 were also found. Regression analysis showed an independent influence of AHI, SpO_2_ min and BMI on a-2-AP. AHI and SpO_2_ min were independent predictors of PAI-1 concentration.

Table 4 presents the results.

**Table 4:** Factors influencing a-2-AP and PAI-1 concentrations determine by multiple linear regression.

Dependent variable	Independent variables	*b*^1^ ^a^	*SE* ^c^	*b* ^b^	*SE* ^c^	P-value
a-2-AP	AHI	0.64	0.09	2.36	0.37	<0.0001
	SpO_2_ min	-0.23	0.07	-1.54	0.49	0.003
	BMI	0.12	0.06	2.16	1.09	0.04
	Model: *R* ^d^ = 0.92, *R*^*2*^ ^e^ = 0.85, corrected *R*^*2*^ = 0.84, p<0.0001					
PAI-1	constant			17.9	6.38	<0.0001
	AHI	0.35	0.13	0.11	0.04	<0.0001
	SpO_2_ min	-0.24	0.13	-0.14	0.07	0.04
	Model: *R*^d^ = 0.53, *R*^*2*^^e^ = 0.28, corrected *R*^*2*^ = 0.25, p<0.001					

a-2-AP, alpha-2-antiplasmin; AHI, apnoea-hypopnoea index; BMI, body mass index; PAI-1, plasminogen activator inhibitor 1, SpO_2_ min, minimum oxygen saturation.

^*a*^ *b*^*1*^ = standardized coefficient of regression,

^b^ *b* = coefficient of regression,

^c^ *SE* = standard error,

^d^ *R* = regression coefficient,

^e^ *R*^*2*^ = coefficient of determination.

## Discussion

Many reports have found that obstructive sleep apnoea favours cardiovascular diseases. The underlying pathomechanisms are not fully understood. The inhibition of fibrinolysis is associated with the development of thrombosis and atherosclerosis, and strong evidence indicates the involvement of plasminogen activator inhibitor 1. Elevated PAI-1 concentration is an important risk factor for both coronary artery disease and cerebral stroke. The role of alpha-2-antiplasmin in the promotion of cardiovascular diseases has not been confirmed thus far. The aim of our study was to evaluate of the impact of OSA on major fibrinolytic inhibitors.

We found significantly higher concentrations of alpha-2-antiplasmin in the plasma of patients with OSA compared with OSA-negative subjects. There was a significant correlation between plasma a-2-AP and BMI, AHI, AI, RTD, DI, mean and minimum oxygen saturation and t90. To the best of our knowledge, this is the first study evaluating the concentration of alpha-2-antiplasmin in patients with OSA.

In recent years, few studies have examined the important regulators of fibrinolysis [32]. Alpha-2-antiplasmin and its deficiency were identified in a report investigating the difficulty in maintaining haemostasis in patients who did not have impaired blood coagulation. A-2-AP was shown to play a decisive role. It is the final component inhibiting the speed and abundance of fibrinolysis through direct plasmin inactivation. Thus, it is clear that increased concentrations will have indirect prothrombotic effects. Currently, however, there is no clinical evidence for this hypothesis as only a few animal studies have been performed thus far.

One of few clinical trials associating alpha-2-antiplasmin with the cardiovascular system was a study by Tomczykowska et al. The authors found significantly lower levels of alpha-2-antiplasmin in the patients with hypertension compared to the control group, suggesting the potential induction of fibrinolysis by hypertension [33]. The results presented by the authors of the above study differed from ours. Statistical analysis did not show a correlation between the concentration of alpha-2-antiplasmin and prevalence of hypertension. Bogdanski et al. provided indirect evidence for the link between a-2-AP and cardiovascular diseases. They analysed the relationship between a-2-AP and obesity. The median value of alpha-2-antiplasmin was significantly higher in the patients with extreme obesity (BMI>40 kg/m^2^) compared to the control group (BMI<25 kg/m^2^). Obesity significantly increases the risk of cardiovascular disease, and therefore, the increased inhibition of fibrinolysis at the level of plasmin may be one of pathomechanisms to explain this phenomenon [34].

Elevated PAI-1 is an independent risk factor of cardiovascular diseases, especially those of atherosclerotic aetiology. The link between PAI-1 and ischaemic stroke has been well studied [35]. Elevated levels of PAI-1 not only positively correlated with the severity of stroke but also greatly increased the failure rate of thrombolytic therapy. Ischaemic heart disease was analogously reported. Swedish, British and Polish reports indicated that elevated levels of PAI-1 should be considered an independent risk factor of coronary artery disease [36].

Our results revealed significantly increased PAI-1 concentrations in the patients with OSA compared to the controls. The highest PAI-1 concentration was detected in the group with severe OSA. Plasma PAI-1 concentrations were significantly correlated with BMI, AHI, AI, RDT, DI, and minimum and mean oxygen saturation. PAI-1 was also higher in patients with hypertension.

In the literature, the relationship between PAI-1 and OSA, particularly in terms of cardiovascular complications, has been studied. Impaired fibrinolysis due to OSA was first demonstrated by Rangemark et al., who analysed the concentrations of the primary fibrinolysis inhibitor, plasminogen activator inhibitor 1 [37]. The results from this study are also consistent with the data published by von Känel et al. The authors evaluated the concentration of PAI-1 in the blood serum of 44 patients with OSA. AHI was the major predictor positively correlated with PAI-1, independent of the others (BMI, age, mean oxygen saturation). There was no significant correlation between the concentration of PAI-1 and gender, blood pressure, haematocrit and smoking cigarettes. Moreover, the authors showed that PAI-1 levels decreased significantly after a 2-week treatment with continuous positive airway pressure (CPAP). The described impact of CPAP therapy on fibrinolytic activity may explain the beneficial effects of this therapeutic approach on the cardiovascular system [19]. In another study, von Känel confirmed a significant correlation between OSA and PAI-1 concentration and demonstrated that the prothrombotic effects depended not only on the number of apnoea and hypopnoea episodes but also on the severity of sleep pattern fragmentation [38].

Moreover, our study also assessed fibrinolytic activity with reference to hypertension. The PAI-1 concentrations were significantly increased in patients with primary and renovascular hypertension compared to the controls (p<0.01) in previous studies. Hypertension not only causes mechanical damage to the endothelium but also inhibits fibrinolysis [39]. Greek researchers analysed the correlations between PAI-1 and early organ damage in patients with OSA. They found higher left ventricular mass index, intima-media thickness as well as microalbuminuria in patients with elevated PAI-1 concentration. [40]. These results raised two questions: whether the increased concentration of endogenous fibrinolysis inhibitors can be used in the future as a marker of endothelial dysfunction and vascular remodelling in the early stages of hypertension, and whether it is related to sleep apnoea? The former question remains unanswered. The answer to the latter question was provided by Spanish researchers. They evaluated fibrinolytic activity by measuring PAI-1 concentrations in patients with OSA, with or without the co-occurrence of hypertension. PAI-1 levels were significantly higher in the patients with OSA and concomitant hypertension compared with patients who were diagnosed with OSA alone. In addition, in both groups, PAI-1 concentrations were significantly higher than in the control group. Obstructive sleep apnoea and hypertension are known to independently increase cardiovascular risk. Considering the fact that sleep apnoea itself favours the development of high blood pressure and is considered by some as its most common secondary cause, OSA additive or hyperadditive effect on PAI-1 levels may significantly increase the risk. The authors’ results would not be surprising if not for the fact that PAI-1 was negatively correlated with AHI in all groups, and the correlation was significant only among the patients with OSA (r = -0.71, p<0.001) [41]. These results contradict the current knowledge on the subject.

The explanation may be found in the research by Shatos et al. They demonstrated that the synthesis and secretion of PAI-1 is carried out under conditions of oxygen deficiency (anoxia); however, PAI-1 concentration decreased significantly during subsequent reoxygenation [42]. Reduced fibrinolytic activity may depend on the total duration of apnoea episodes and not on their number (the higher number of apnoeas, the greater number of reoxygenation). This hypothesis requires further study.

Significantly higher concentrations of PAI-1 and alpha-2-antiplasmin in plasma of patients with obstructive sleep apnoea suggest the presence of increased prothrombotic activity in this group of patients. This activity increases with the severity of the disease. Obstructive sleep apnoea may increase the risk of cardiovascular complications via the intensification of prothrombotic activity.

The study has several limitations. It was conducted in one center. Small number of patients in the study with predominance of men is the major one and it is mostly due to prespecified eligibility criteria. Study was limited also by use of screening polysomnography (a type 3 device according to American Academy of Sleep Medicine) for OSA diagnostics, which does not allow to calculate AHI per hour of sleep (only per hour of examination). Additionally, alpha-2-antiplasmin and PAI-1 concentration was determined by single laboratory method (ELISA).
